# Cell killing and resistance in pre-operative breast cancer chemotherapy

**DOI:** 10.1186/1471-2407-8-201

**Published:** 2008-07-21

**Authors:** Paolo Ubezio, David Cameron

**Affiliations:** 1 Biophysics Unit, Department of Oncology, Istituto di Ricerche Farmacologiche "Mario Negri", Via La Masa 19, I-20156 Milan, Italy; 2 Department of Oncology, Edinburgh University, Western General Hospital, Crewe Road South, Edinburgh EH4 2XU, UK

## Abstract

**Background:**

Despite the recent development of technologies giving detailed images of tumours *in vivo*, direct or indirect ways to measure how many cells are actually killed by a treatment or are resistant to it are still beyond our reach.

**Methods:**

We designed a simple model of tumour progression during treatment, based on descriptions of the key phenomena of proliferation, quiescence, cell killing and resistance, and giving as output the macroscopically measurable tumour volume and growth fraction. The model was applied to a database of the time course of volumes of breast cancer in patients undergoing pre-operative chemotherapy, for which the initial estimate of proliferating cells by the measure of the percentage of Ki67-positive cells was available.

**Results:**

The analysis recognises different patterns of response to treatment. In one subgroup of patients the fitting implied drug resistance. In another subgroup there was a shift to higher sensitivity during the therapy. In the subgroup of patients where killing of cycling cells had the highest score, the drugs showed variable efficacy against quiescent cells.

**Conclusion:**

The approach was feasible, providing items of information not otherwise available. Additional data, particularly sequential Ki67 measures, could be added to the system, potentially reducing uncertainty in estimates of parameter values.

## Background

Mathematical modelling of cancer and cancer therapy has been attempted on all scales. There have been interesting examples ranging from the microscopic levels of single molecular interactions and protein network modelling of specific cellular functions [1], to cell cycle and *in vitro *cell proliferation, simulating the time course of cytostatic and cytotoxic effects of drugs [2-4], up to the macroscopic level considering tumour growth *in vivo *[5-13].

Mathematical models differ also in the way they use experimental data, some being more theoretical, trying to explain the general behaviour of a biological system, and others aiming to fit specific data sets.

At the level of *in vivo *tumour growth, a typical datum is the time course of tumour volumes, measured (by callipers or *in vivo *imaging) in animals in the pre-clinical stage of drug development or in humans. Because these are relatively simple datasets, simple descriptive models of tumour growth have been used. For instance Skipper adopted a model of exponential growth, defined by the number of tumour cells at the start of the observation period and by the doubling time [14]. This model was appropriate to describe leukaemia developing in mice. The effect of a cytotoxic drug was described by the fraction of cells killed at each administration, so the complete model (tumour growth + treatment) had only three parameters.

Norton and Simon improved Skipper's model and used the Gompertz function to describe a continued decline of the growth rate as the tumour mass increases [15]. This shape fits many experimental time courses of solid tumours, particularly in animals, where the whole natural history of the tumour can be followed. Reduction of drug efficacy in massive tumours was described by a simple link between the fraction of surviving cells at each treatment and the growth rate. Similarly, Goldie and Coldman [16] and others [8,10] tackled the issue of drug resistance using the probability to spontaneous mutation(s) towards a resistant phenotype (i.e. with a single parameter).

Of course all these authors were aware that tumour growth and response to a drug are very complex phenomena, with many interacting factors, from molecules to environmental constraints. For instance, the dynamics of the response to a drug challenge was recently modelled using no fewer than six time-dependent parameters, associated with cell cycle block and cell killing in each phase of the cell cycle [2]. In that work, considering a single population of tumour cells growing *in vitro *in the best environmental conditions, a less rich model was unable to fit all available data (a dataset including measures with different techniques). Thus, in a certain sense, the success of simple models at the higher *in vivo *level is probably a consequence of the scanty datasets currently available. On the other hand, using complex models with many parameters to describe tumour growth seems a purely theoretical exercise, not giving any useful additional clinical insights if – as is often the case-those same experimental time courses are satisfactorily fitted (taking into account the poor precision of the data) by simple polynomial or exponential functions.

In this paper we propose an intermediate approach. We adopt a richer data set from patients with breast cancer receiving pre-operative chemotherapy, including a measure of proliferation (Ki67 staining), and use an interpretative (phenomenological) model to fit the data. This new model is basically different from the previously reported model [17,18] of breast cancer response to therapy. The "old" model attempted to estimate the values of cell kill and resistance parameters that were consistent with the overall decrease in volume of individual tumours, simplifying with a single growth equation the net result of the interplay between cell cycle, quiescence and loss.

Differently from models based on simple descriptive functions (like exponential and Gompertz), the new model was based on that underlying interplay. Because of the nature of breast cancer, where only a fraction of the cells are actually cycling (the fraction estimated by the measure of Ki67), an essential feature in the new model is the consideration of quiescence [9,19,20], together with proliferation and cell loss, exploiting the results of the mathematical theory of age-structured cell populations with a quiescent compartment [21,22]. As a consequence, the response to treatment was modelled taking into account of the different effects against cycling and quiescent cells. In addition, variants of the basic model with increasing complexity were considered, starting from a model neglecting the resistance up to a model including resistance and the shift of treatment efficacy during the course of treatment. We adopted the principle of parsimony, accepting the simplest model when the inclusion of additional parameters did not significantly improve the fit. We investigated not only how well this model fitted the data of several patients, but also the precision of the estimates of parameters. Taking that precision into account, the values of parameters characterising treatment efficacy (killing of cycling and of quiescent cells, drug-resistant fraction) were categorized and further analysed for association and correlation with common biological markers. Eventually, we will discuss which additional data would be useful to improve the precision of parameters estimate.

## Methods

### Patients and histology

Thirty-five patients with large operable breast cancers (stages T_2 _or T_3_) received preoperative chemotherapy based on cyclophosphamide 1 g m^-2 ^and doxorubicin 50 mg m^-2 ^every three weeks. Eleven patients already had hormone therapy for up to three months which had not induced any tumour shrinkage.

Histological confirmation of invasive breast cancer was obtained by wedge biopsy of the primary tumour or a palpable axillary node, from which material was also made available for determination of the estrogen receptor (ER) concentration and Ki67 as a measure of tumour cell proliferation. ER concentration was measured by the dextran-coated charcoal (DCC) method. Ki67 antigen was revealed by immunohistochemical staining with antibody MIB1 (Europath Ltd, Cornwall, UK) diluted x50. Reactivity was detected by an ABC-peroxidase-antiperoxidase (PAP) method providing the percentage of Ki67-positive cells (%Ki67+) at the start of treatment. Mean %Ki67+ measured in this set of tumours was 37% (standard deviation: 24%).

Tumour volume was estimated weekly for at least six weeks, by measuring two orthogonal diameters with callipers and applying the ellipsoid volume formula, assuming the third dimension as the average of the other two. The tumour volumes measured on day 0 ranged from 22 cm^3 ^to 204 cm^3^, mean 70 cm^3^, median 58 cm^3^; the detection limit was 0.1 cm^3^. The number of positive axillary nodes (NOD) and the clinical and pathological response were assessed at the time of surgery.

Clinical response was classified according to the UICC criteria [23]. Clinical complete remission (CR) was defined as the disappearance of all palpable tumour deposits and partial remission (PR) as a more than 50% reduction of tumour volume. Tumour reduction less than 50% or an increase up to 25% was scored as stable disease (SD). An increase of more than 25% was designated as progressive disease (PD).

Pathological complete remission (pCR) was defined as a tumour with no residual microscopic disease in either the breast or ipsilateral axillary lymph nodes.

All patients in this study were treated within the context of a clinical trial which had been approved by the regional ethics committee, and for which patients gave informed consent. Subsequently further ethics approval was obtained from the same committee to cover a broad range of research projects, including that reported herein on the tumour tissues, which did not require patients to be explicitly re-consented.

### Model

The program combined the three models of tumour growth, treatment and resistance. The underlying mathematics is reported in Appendix 1.

### Tumour growth model

**Figure 1:**
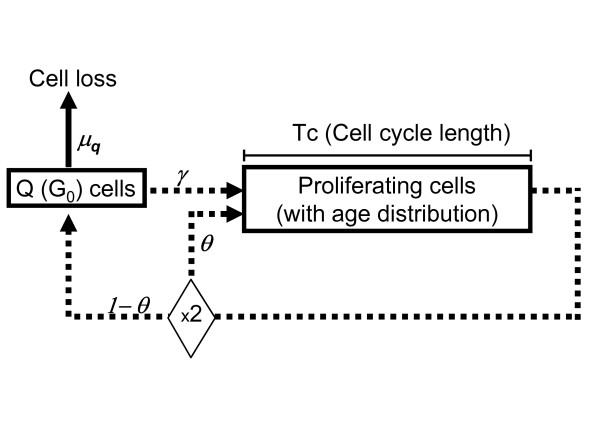
The model of tumour growth.

The model included a compartment of quiescent cells, and a compartment of cycling cells with age structure (Figure 1). Cycling cells spent a time *Tc *in the cell cycle before division. After division, newborn cells either re-start the cycle, with probability *θ*, or enter the quiescent compartment, with probability 1-*θ*. Quiescent cells may die (with rate *μ*_*q*_, so that *μ*_*q*_dt is the probability to die in the time interval dt) or re-enter the cycle, with rate *γ*. We assumed that most cells become quiescent before dying, and the probability of "natural" death (in the sense of "not induced by a treatment") was applied only to quiescent cells

Within this model, in order to identify univocally the tumour growth characteristics we need to specify four measurable (at least in principle) macroscopic quantities: the doubling time (*Td*), *Tc *(or *Tpot*, the potential doubling time), the growth fraction (*GF*) and *γ*. The other parameters (*θ *and *μ*_*q*_) rare dependent on these four quantities (see Appendix 2).

### Treatment model

The main feature of the model is the drug's different effects on cycling and quiescent cells. The basic parameters are:

*Sp*: the fraction of cycling cells surviving a single drug administration.

*Sq*: the fraction of quiescent cells surviving a single drug administration.

To reproduce the (slow) process of disruption/elimination of killed cells, we adopted the method described by Simeoni *et al*. [11], successfully applied to experimental tumours. In brief, dying cells stop proliferating and pass through three stages, with progressive degrees of damage, before they are definitively lost. The passage from one stage to the next is mathematically described by a rate constant *k*, which thus adds to the parameters of the model (see Appendix 1).

### Resistance model

Two distinct modalities of resistance were compared: 1) "Initial Resistance", when a fraction of the cells was already resistant to a drug at the start of treatment; 2) "Induced Resistance", when a fraction of sensitive cells surviving treatment became resistant. Each modality was modelled by a single parameter, either:

*IniR*: the fraction of cells initially resistant to the drugs, or

*Rind*: the fraction of sensitive cells becoming resistant after a single treatment.

### Fitting procedure and sensitivity analysis

Eventually, merging the three models of tumour growth, treatment and resistance and including the tumour volume at the beginning of treatment (*V*_0_), we have a model with nine independent parameters: *V*_0_, *Td*, *Tc*, *GF*, *γ*, *Sp*, *Sq*, *k*, *IniR *(or *Rind*).

In order to reduce the number of parameters for fitting, we assumed a specific type of tumour growth, exploiting the measure of Ki67 as an estimate of the growth fraction, and found the parameters of cell kill and resistance by an optimisation procedure. A constrained, non-linear fitting procedure was used, maximising the likelihood function of the logs of tumour volumes, with Gaussian distribution of data errors, taking their standard deviation as a parameter. The following constraints were used:

0.7·V_0measured _<*V*_0 _< 1.3·V_0measured_: this assumes that the measure of volume at t = 0 had 30% precision;

0 ≤ *IniR *(or *Rind*) < 1: these are probabilities;

*Sp *<*Sq*: this assumes that cycling cells are more sensitive to treatment than quiescent cells;

*Sp *> 0.001: this assumes that no single treatment will kill more than 99.9% of sensitive cycling cells;

*Sq *≤ 0.98: this assumes that no single treatment will kill less than 2% of sensitive cells (otherwise cells are defined as "resistant");

0.5 ≤ *k *< 1: the minimum value of *k *(0.5) produced a delay in the loss of dead cell such that 14 days are needed to lose 99% of the dying cells. When *k *approaches 1 almost all dead cells are lost in three days.

This procedure was repeated assuming other types of tumour growth, covering all possible combinations of doubling time (either short – 30 days-, intermediate – 150 days-, or long – 10000 days), cell cycle durations (2, 5 or 8 days) and recycling rate (0 or 0.01), consistent with the value of the growth fraction (see Appendix 2). The maximum-likelihood of the fits obtained with all growth types were compared and the one with highest likelihood (L_best_) was assumed as best fit for the patient's tumour time-course. The likelihood ratio test statistics (LRTS) was used to compare a fit with a given set of parameters "X" with the best one (LRTS = 2(log(L_best_) - log(L_X_)). As LRTS follows a chi-square distribution, X was considered equivalent to the best when LRTS < χ^2^_0.05,1_. Similarly, likelihood-based 95% confidence intervals for each parameter were obtained by raising or lowering its value until L was reduced to the value of log(L) = log(L_best_) - χ^2^_0.05,1_/2 [24,25]. The overall range of variability of a parameter was calculated by joining up the confidence intervals obtained with all tumour growth models equivalent to the best.

The whole procedure was repeated using three models of treatment and resistance, at increasing levels of complexity. The lowest level (Level I) considered no resistant cells and the same cell survival at each drug administration, with a difference in sensitivity between cycling and quiescent cells (parameters: Sp and Sq). Level II included a parameter measuring drug resistance, in two variants describing either initial or drug-induced resistance (parameters: Sp, Sq, IniR (Rind). Level III included a sensitivity shift after some chemotherapy cycles (parameters Sp, Sq, Sp2, Sq2, describing the fractions of surviving cells respectively in the first and second period).

### Implementation

All analyses were done with a computer program using Microsoft Excel with its standard features (Visual Basic and Solver). With a user-friendly interface, an automatic graphic output of the simulation curve with data is provided at each change of parameter values. The program ("PAOTHERAPYA") is available for non-commercial purposes in Additional file 1.

## Results

### Adequacy of the models

Data were fitted using the principle of parsimony, adopting a lower-level model when a higher level did not significantly improve the fit. About half of time-courses had a regular decreasing trend and were fitted simply by applying the same fraction of surviving cells (Sp, Sq) at each therapy cycle (Level I). In 25% of cases the efficacy declined during therapy, and the data were fitted assuming a fraction of cells resistant to treatment (Level II). In another 25% of cases the efficacy appeared to increase, after 20 (5 patients) or 60 days (4 patients), and the data were fitted with a sensitivity shift, with two phases characterised by different surviving fractions (Table 1).

**Table 1:** Summary of the models required for fitting.

**Model type**	**Resistance**	**Sensitivity shift**	**Parameters**	**Cases**
Level I	NO	NO	Sp, Sq (1)	17/35
Level II	YES	NO	Sp, Sq, IniR (2)	9/35
Level III	NO	YES	Sp, Sq, Sp2, Sq2 (3)	9/35

(1) fraction of cycling (Sp) and quiescent (Sq) cells surviving a single cycle of therapy.

(2) fraction of cells resistant to treatment (calculated at time zero)

(3) fraction of cells surviving a single cycle of therapy in the first (Sp, Sq) and second (Sp2, Sq2) phase sensitivity.

**Figure 2:**
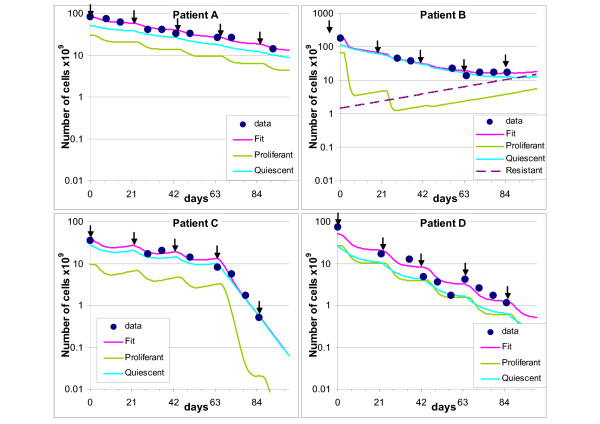
**Examples of fitting**. Representative examples of fitting (continuous magenta line) of experimental time courses (dots). The arrows indicate treatment times. Outlines of cycling (green line) and quiescent (light blue line) cells predicted by the model are also reported. Panel A: fitting with a level I model, with *Sp *= 0.68 and *Sq *= 0.98 (*k *= 0.50). Panel B: fitting with a level II model, including a subpopulation of cells resistant to treatment (dashed line). The fit was obtained with *Sp *= 0.04, *Sq *= 0.90 and *IniR *= 0.01 (*k *= 0.83). Panel C: fitting with a level III model, with an increase of efficacy from the fourth cycle. The fit was obtained with *Sp *= 0.43 and *Sq *= 0.98 up to day 60 and *Sp *= 0.004 and *Sq *= 0.98 thereafter (*k *= 0.50). Panel D: an example of unsatisfactory fit, with *Sp *= 0.34 and *Sq *= 0.98 (*k *= 0.50).

Representative data and fits are shown in Figure 2. Panel A shows a time course of data (dots) together with the fitting using a level I model (magenta line). The arrows indicate the treatment times. The outlines of cycling and quiescent cells predicted by the model are also reported. In this case the model predicted almost 30% cycling and few percent quiescent cells killed at each treatment cycle, regularly up to the last one, with no onset of resistance and no increase of sensitivity.

Panel B shows an example of fitting with a level II model, including a subpopulation of cells resistant to treatment (dashed line). The model predicted about 95% cycling and 10% quiescent cells killed at each treatment, but this efficacy cannot be maintained after the fourth cycle. In order to fit the last points of the time course, the model predicted that 1% of the cells were initially resistant to the therapy and this subpopulation of cells eventually became prevalent (more than 80% resistant cells by day 100).

Panel C shows an example of fitting with a level III model, with an increase of efficacy from the fourth cycle. The model predicted that about 60% of cycling cells were killed in each of the first three cycles, but the fourth killed more than 99%. The efficacy of treatment against quiescent cells remained low.

Best fits for the data of 31/35 patients had a discrepancy between simulated tumour volume and datum below 30% (calculated as the average of all time points), and 26/35 were below 20%. Panel D shows one of the four instances where a less satisfactory fit was obtained. The presence of one or two (probable) outliers in the time course explains the poor score in these cases. They were not excluded from subsequent analyses, because the simulation caught the general pattern of the outline in these cases too.

The performance improves on that obtained with the previously published model [18], with no distinction between cycling and quiescent cells. With the latter simplified model the fittings were always worse, and only in 19/35 instances was the average distance from data below 30%.

The simulation program permits extrapolation of the time course of the tumour volume up to a fixed time, in order to compare patients at the same time-point. Assuming day 100 as end-point, the model suggested that the volume was reduced at least 50% in all 26 non-resistant cases, and by more than 90% in 16/26 instances.

### Estimate of cell kill

**Figure 3:**
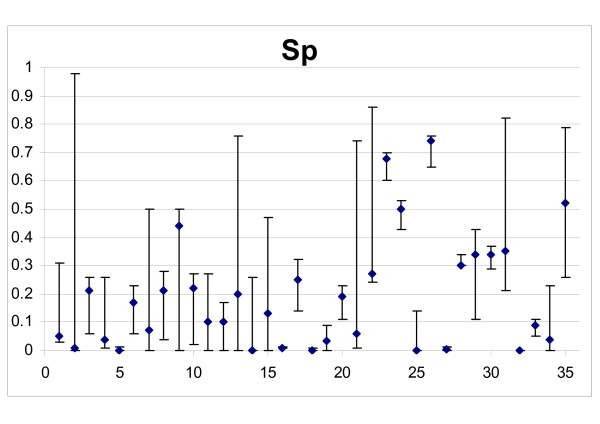
**Drug efficacy on cycling non-resistant cells of all tumours**. Abscissa: patient ID numbers; Ordinate: surviving fraction (*Sp*) in the best fits, with 95% confidence intervals.

Figure 3 shows the best fit estimates of drug efficacy on cycling non-resistant cells of all tumours, in terms of the surviving fraction Sp, with their 95% confidence intervals. Although in some instances the confidence intervals were very wide (in 6/35 more than 0.5), in most cases the data were informative enough to indicate that more than 50% of cycling cells were killed at each chemotherapy cycle. A score was assigned to cell killing, from 1 to 5, indicating "very low" (Sp > 0.5), "low" (0.25 < Sp ≤ 0.5), "medium" (0.10 < Sp ≤ 0.25), "high" (0.01 < Sp ≤ 0.1) and "very high" (Sp ≤ 0.01) drug efficacy. Taking confidence intervals into account, the score was reduced by "1" when the range exceeded the limit of the previous category. When the confidence range was 0.001 – 0.98, Sp was considered not detectable (ND) and no score was assigned. Sp was not detectable in only one case.

Table 2 shows the cases in the five categories. Cell killing was high or very high in 31.3% detectable patients, medium in 35.3%, low or very low in the remaining 32.3%.

**Table 2:** Frequency of the scores of killing of cycling cells per treatment in the dataset.

**Score Sp**	**freq**	**%**
1	5	14.7
2	6	17.6
3	12	35.3
4	6	17.6
5	5	14.7
ND	1	

**Figure 4:**
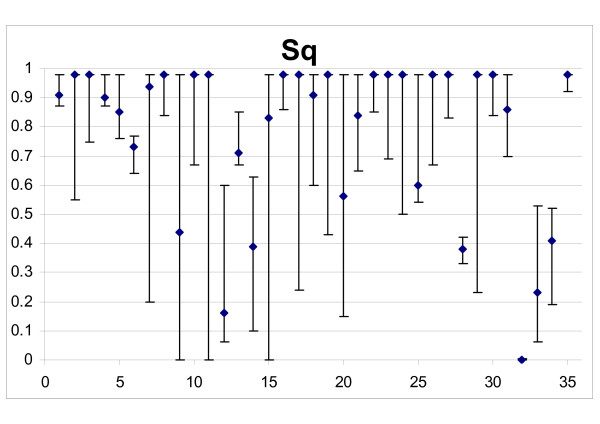
**Drug efficacy on quiescent non-resistant cells of all tumours**. Abscissa: patient ID numbers; Ordinate: surviving fraction (*Sq*) in the best fits, with 95% confidence intervals.

The survival of quiescent cells (Sq) was estimated with lower precision than Sp (Figure 4) and in ten cases (28.6%) confidence intervals were > 0.5. Three cases were not detectable (confidence range: 0.001 – 0.98).

Although best fits suggested values above 0.9 (90% quiescent cells surviving a single treatment) in 18/32 detectable patients, Sq was below 0.5 in six cases and in another six cases the best fit was above but the lower limit of the confidence interval was below 0.5. Thus, the results suggest that killing of quiescent cells was important at least in some – not rare-instances.

Comparing the model results with the clinical/pathological response, 8/10 cases identified as responders (pCR or CR) were coherently classified using the cycling cell killing scores 4 or 5, the other two with score 3. Three cases scored by the model with high cell killing were only PR: two had a late sensitivity shift and the third experienced a 2-log mass reduction.

### Resistance

**Figure 5:**
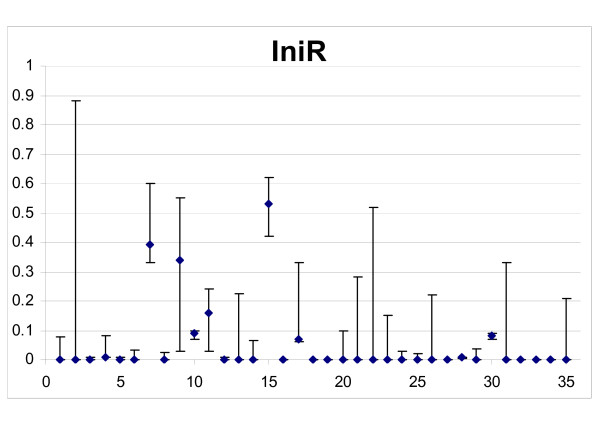
**Evaluation of resistance**. Abscissa: patient ID numbers; Ordinate: resistant cells, as a fraction of the initial cell number (*IniR*) in the best fits, with 95% confidence intervals.

A subpopulation of resistant cells was found in 9/35 instances, with confidence intervals all above zero and fitted using a level II model (Figure 5). However, in eight other cases resistance could not be precisely evaluated and data were compatible with no resistance as well with the presence of more than 10% resistant cells. This reflects the difficulty of distinguishing "low sensitivity" from "resistance" with the available data. Skipping the ambiguous cases, it resulted that resistance was present and reduced treatment efficacy in 9/27 i.e. 33.3% cases. For convenience, therefore we will consider three groups of resistance:

group 1, 18 cases where a resistant subpopulation was excluded (formally: best fit with *IniR *= 0 (model type I) and maximum *IniR *< 0.1 at the 95% confidence interval);

group 2, "undetermined resistance", comprising the eight ambiguous cases above;

group 3, nine cases where the presence of resistant cells was demonstrated by a significantly better fit.

The nine cases in group 3 were further analysed with an alternative model where no resistant cells were initially present but resistance was induced by treatment. This enabled us to fit the data with similar precision (not shown). Table 3 shows the range of values of the resistance parameters *IniR *(the fraction of cells initially resistant, used in the standard model) and *Rind *(the fraction of sensitive cells becoming resistant after a single treatment, used in the alternative model). With the first assumption, resistant cells amounted, on average, to 19% of the tumour at the beginning of therapy. However, assuming resistance induced by treatment, 33% of surviving cells, on average, must become resistant as a consequence of a single drug challenge, which is probably unrealistically high.

**Table 3:** Resistance parameters.

	**IniR**	**Rind**
Average	0.19	0.33
Minimum	0.01	0.02
Maximum	0.53	0.85

Statistic of best fit resistance parameters in a model where cells are initially resistant to therapy (*IniR*) and in an alternative model where resistance is induced by treatment (*Rind*), in the subset of tumours where the presence of resistant cells was demonstrated.

### Resistance and cell kill related to estrogen receptor content and number of positive nodes

**Figure 6:**
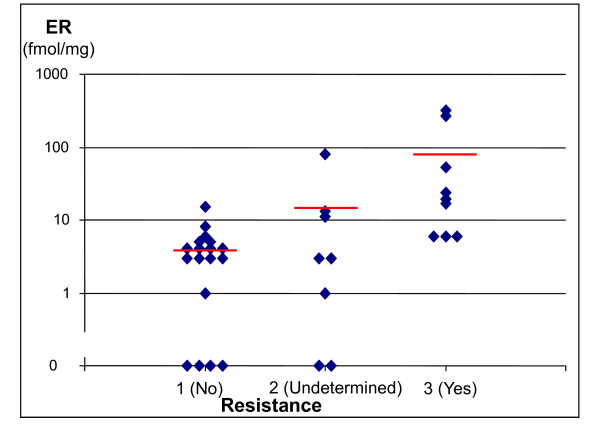
**ER content in the three resistance groups**. Ordinate: ER content (fmol/ml); Abscissa: group 1: no resistance; group 2: resistance undetermined (data were fitted without resistance, but models including more than 10% resistant cells gave equivalent fits); group 3: resistance required (models including resistance significantly improved the fit). Short horizontal lines represent the average value in each resistance group.

Figure 6 shows ER values in the three groups of patients based on the resistance parameter. Average ER value in group 1 (no detectable resistance) was 4 fmol/mg (maximum 15), compared to 80 in group 3 (detectable resistance), with ER > 15 in 6 out of 9 instances. In group 2, i.e. the 8 patients with "undetermined" resistance, average ER was 14 fmol/mg (≤ 15 fmol/mg in 7/8 patients).

**Figure 7:**
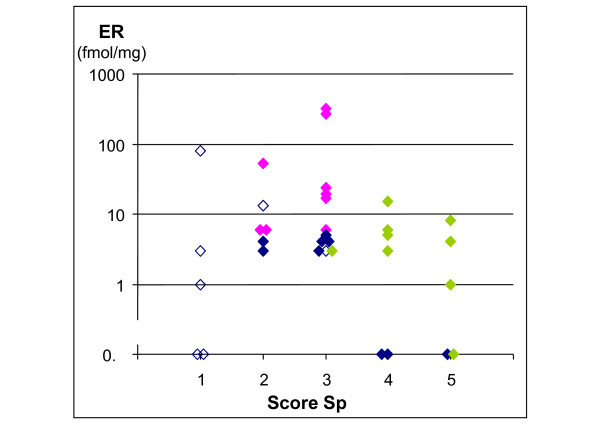
**ER content in the five efficacy groups**. Ordinate: ER content (fmol/ml); Abscissa: Sp score (score 1: lowest cell kill of non-resistant cycling cells score 5: highest cell kill). Colours refer to the type of model of the best fit. Blue: Level I (without resistance. Open symbols: resistance undetermined; closed symbols: no resistance); Fuchsia: Level II (with resistance); Green: Level III (without resistance, with biphasic efficacy, Sp calculated on the more effective phase).

Figure 7 reports ER values in relation to treatment efficacy on non-resistant cells, as measured by the score of the parameter Sp. ER was unrelated to the strength of cell killing. Dividing the cases according to the type of model required for fitting showed that the cases fitted with each model clustered in different regions of the plot. Cases fitted with model type III, including biphasic efficacy, clustered in the region of highest cell kill (scores 4–5) and low (<15) ER; model type II, including resistance, clustered in a region of intermediate efficacy (scores 2–3) and high ER values; model type I without resistance was present in the region of intermediate or high efficacy and low ER, while model type I but with undetermined resistance (with the criteria specified above) grouped in the low sensitivity region (score 1), with a spread of ER values.

Among patients which were given chemotherapy after not responding to conventional hormone therapy, those with ER > 20 fmol/mg at the start of chemotherapy were classified as "resistant" (four) or "undetermined" resistance (one), while the six patients with ER < 20 fmol/mg all responded to chemotherapy (not shown).

Table 4 shows the relationship between resistance and the number of positive nodes. Ten of the 18 patients with no detectable drug resistance were node-negative and only three had more than two positive nodes, while in group 3 two out of nine were node-negative and six had more than two positive nodes.

**Table 4:** Resistance vs. Number of positive nodes. Patients' subsets on the basis of the presence of resistant cells and of the number of positive nodes.

	**Nr. of nodes**			
**Presence of resistant cells**	**0**	**1–2**	**>2**	*Total pts:*

No (group 1)	10	5	3	*18*
Uncertain (group 2)	3	1	4	*8*
Yes (group 3)	2	1	6	*9*

*Total pts:*	*15*	*7*	*13*	*35*

## Discussion

Despite the increasing knowledge of the molecular biology of tumours and their interactions with anticancer drugs, less is known about measuring the benefits of treatments in patients in terms of percentages of tumour cells killed and the proportion that is resistant. This study addresses these two questions during classical neoadjuvant chemotherapy in early breast cancer by interpreting a series of time-course data of tumour volumes, plus the measure of Ki67, using basic models of tumour growth, treatment and resistance. By fitting the tumour outlines, with sensitivity analysis, the range of cell killing and resistance parameters were estimated.

The importance of proliferation markers as predictors of treatment outcome in breast cancer management has been previously investigated, with controversial results [26]. A single marker, like Ki67 or apoptotic index, provides only a partial view of the underlying tumour kinetics, which is based on the interplay between cell cycling, quiescence and cell death (by apoptosis or other mechanisms). Furthermore, the different effects of treatment on quiescent and cycling cells, besides the presence of drug-resistant subpopulations, although theoretically recognised, is seldom taken into account in the analysis of outcome or in the design of chemotherapy regimens. This paper takes a step towards including all these variables in a comprehensive reconstruction of the dynamics of the response to treatment. This mathematical reconstruction extends the conventional assessment of clinical and pathological responses, which cannot recognise the details of a partial remission, to provide clues on a patient's sensitivity which are potentially useful to decide extension or changes of treatment.

The mathematical model used in this study performs better in reproducing the volume decrease during treatment than previous approaches [18] and explicitly includes cell cycle, quiescence and loss. This made the model more complex but also more realistic and allowed connections with the kinetic quantities that are – at least in principle – measurable *in vivo*, namely the doubling time, the potential doubling time and the growth fraction. We could then incorporate the measure of Ki67, as an estimate of the individual tumour growth fraction.

Because in most breast tumours only a minority of cancer cells are actually Ki67-positive and cycling [27], it seemed mandatory to include quiescent cells in the model [28]. As a consequence, two parameters of killing, one for cycling and the other for quiescent cells, were used in the fitting. However, because the data, including the Ki67 measure, were still not sufficient for an unequivocal assessment of tumour growth in the absence of treatment, we adopted a conservative strategy, examining a range of possibilities for each patient from fast to slow tumours. We repeated each fitting with 18 different growth models, combining representative values for each kinetic parameter. That choice was pursued with a conservative sensitivity analysis, including all growth models, to assess the uncertainty of estimates of parameters describing cell kill and resistance.

Our analysis identified different patterns of response during chemotherapy. A subset of patients was characterized by unequivocal presence of resistance, as the goodness of fit was significantly improved with the inclusion of a subpopulation of cells unaffected by the treatment. For this group of "resistant" patients, the average ER level was more than ten times higher than in the remaining patients. Conversely. six out of the seven patients with ER > 15, were in the "resistant" group, and the seventh had "undetermined" resistance. Instead the association between resistance and number of positive nodes was less striking. The association of the response to treatment with negative ER status has been reported by many others [29,30], but the association ER with the presence of resistant cells could not be directly demonstrated before. Instead, the cell kill parameter was not associated to ER status in our analysis, suggesting that drug efficacy on sensitive cells was unrelated to ER. One possible mechanism explaining the relationship between resistance and ER status is the co-expression of ER and the Bcl-2 proto-oncogene [31], which would be expected to reduce the rate of apoptosis.

A second subset of patients objectively identified by the model was characterized by a sensitivity shift, tumours becoming more drug-sensitive after the first cycle or even later. In this subset of patients the tumour volume at the beginning of treatment was not particularly high and the sensitivity shift occurred after a moderate debulking (not shown). Possibly in some instances the true cell number reduction was not reflected in a volume decrease in the first weeks so the drug effect was initially underestimated. Sensitivity in this group was therefore scored on the basis of the killing parameter estimated in the second, more effective, phase. This was a very sensitive subset of patients: eight out of nine had the highest cell killing scores (4 or 5 for cycling cells), reached in only three of the 25 patients without sensitivity shift (see Figure 7). The observation that sensitivity can increase after up to two months of low treatment efficacy, raises questions about the practice of automatically changing therapy after a poor initial response, and about the optimal number of cycles in some patients.

Considering the subset of patients characterised by high cell killing among cycling cells (score 4 or 5), treatment was also effective against quiescent cells in half cases, indicating the drugs were not exclusively active on cycling cells. However in the other half quiescence limited drug efficacy. These patients might have benefited from strategies aimed at mobilising quiescent cells, or suitably spaced cycles in order to avoid repeatedly hitting tumours with few target cycling cells, or from some entirely different strategy.

This study found that, by combining the time course of tumour volumes with a measure of Ki67, the estimates of parameter values were precise enough to permit a potentially useful and informative classification of the responses. However, that precision would be improved by using richer datasets, including additional measures of proliferation and cell death, not available for the present group of patients. For instance a direct estimate of Td or Tpot would have limited the uncertainty of the growth model. However, Tpot, is nowadays rarely measured in clinical routine and a measure of Td is probably even less feasible, requiring a second volume measurement before the start of treatment, e.g. at diagnosis, or during discontinuation of treatment, when planned in particular situations.

For what concerns cell death, apoptosis is usually measured on histopathological samples, using morphology, caspase activation or the terminal transferase dUTP nick end labelling (TUNEL) technique. This gives at least a qualitative assessment of whether some cells are killed, but unfortunately the relationship between the measured percentage of apoptotic cells and the percentage of killed cells remains unclear, as we do not know how long apoptotic cells remain visible. Thus, such measures of apoptosis are not valid quantitative measures of cell kill.

Another way to improve the precision is with a second evaluation of the growth fraction at the end of therapy, by Ki67 measures on the surgical specimen, or during therapy by fine-needle aspirates or core-cut biopsies. These measures are currently taken in trials of new neoadjuvant therapies, to detect changes in Ki67 as a marker of the response, in the absence of other strong short-term endpoints [32,33]. The mathematical approach would allow to interpret these data within a formal framework including all actors in play, i.e. cycling, quiescent and resistant cell subpopulations. In this respect, the effort made here to assess an objective method of parameter estimate could be exploited, and model's kill rate categories, as well as detection of resistance, could become useful markers of outcome, at an intermediate level between the detection of inhibition of a target and the evaluation of survival.

Finally, new imaging techniques – CT, NMR, PET or even optical – are potential sources of data that can be interpreted by the model, as these techniques give more precise estimates of the overall tumour mass, and permit time-course evaluations for otherwise undetectable or unmeasurable tumours. However, additional research and modelling will be required to connect specific functional measures to the basic "phenomena" of proliferation, quiescence and loss which are the core of our representation of the tumour.

## Conclusion

We presented here a new approach to the evaluation of chemotherapy using a mathematical model to interpret the time courses of tumour mass during treatment, including measures of the proliferative activity by Ki67. The model gives as output objective estimates of the fraction of cells killed by each cycle of treatment and of the fraction of resistant cells. The approach was proven feasible, providing items of information not otherwise available in pre-operative breast cancer chemotherapy. Additional data, particularly sequential Ki67 measures, could be added to the system, potentially reducing uncertainty in estimates of parameter values.

## Competing interests

The authors declare that they have no competing interests.

## Authors' contributions

PU performed modelling, data analysis and drafted the manuscript; DC was involved in study design, performed clinical database management, contributed to data interpretation and manuscript preparation. All authors have read and approved the final manuscript.

## Appendix

### Appendix 1. The models of tumour growth, cell killing and resistance

The equations of the model of tumour growth shown in Figure 1 were thoroughly described by Bertuzzi, Gandolfi *et al *[21], thereafter referred as BG theory, assuming balanced exponential growth. From BG theory, neglecting for simplicity the distinction between G_1_, S and G_2_M phases and assuming "natural" cell loss only from quiescent cells, the density of proliferating cells of age "a" at time "t" is given by the formula

(1) n_p _(a, t) = C e^*bt *^e^-*ba*^

where C is a suitable constant and *b *is the growth rate constant (*b *= ln(2)/*Td*).

By integration on age over the cell cycle time *Tc*, we obtain the number of proliferating cells:

(2) Np (t) = C e^*bt *^(1-e^-*bTc*^)/*b*

At a given time t, cells of age *Tc *end their cycle by division. Considering eq 1, their density is:

(3) g(t) = C e^-*bTc *^e^*bt *^

It is useful to define the quantity u = g(t)/Np(t). In this way, using eq. 2 and eq. 3 we have:

(4) u = g(t)/Np(t) = *b*/(e^*bTc*^-1)

Notice that in the BG theory the following more general version of eq. 4 holds:

(4bis) u = g(t)/Np(t) = *β*/(e^*βTC*^-1)

where *β *= *b *+ *μ *(*β *= *α *+ *μ *in the original notation used in [21]) and *μ *is the cell loss rate of proliferating cells (assuming *μ*_G1 _= *μ*_S _= *μ*_G2M _= *μ *and thus *β*_G1 _= *β*_S _= *β*_G2M _= *β*). Eq. 4 bis can be readily demonstrated by combining the BG equation giving g(t) (eq. 13 in [21]) with BG equations giving Np(t) (eqs. 20 and 21 in [21], remembering that Np(t) = N_G1_(t) + N_S_(t) + N_G2M_(t)).

Eq. 4 bis reduces to eq. 4 when neglecting "natural" cell loss in proliferating cells (*μ *= 0).

Summarizing the theory, we can say that in a small time and age interval (dt = da):

i) u Np(t) dt cells divide originating 2·u Np(t) dt newborn cells, where u is given by eq. 4;

ii) *θ*·2·u Np(t) dt newborn cell enter the proliferating status with zero age and

iii) (1-*θ*)·2·u Np(t) dt newborn cell enter the quiescent compartment.

Noticeably, the age-dependence of the theory is no more explicit in these relationships, being conveyed by the quantity "u", which depends only on *Tc *and *Td*.

Considering non-infinitesimal time intervals, Δ, the number of dividing cells in the interval (t-Δ, t) is given by: $\int_{t-\Delta }^{t}\text{u Np(}\tau \text{) d}\tau$ = u·Np(t-Δ)·$\int_{0}^{\Delta }{\text{e}}^{\text{b}\tau }\text{ d}\tau$ = u Np(t-Δ)·z where:

(5) z = (e^*b*Δ^-1)/*b*

Similarly, the number of quiescent cells becoming proliferating or dying in the interval (t-Δ, t) is *γ*·Nq(t-Δ)·z and *μ*·Nq(t-Δ)·z respectively.

The above definitions and equations allowed to simulate tumour growth by finite differences with time step Δ (Δ = 1 day in the simulations presented in this paper), calculating the numbers of cycling (Np(t)) and quiescent (Nq(t)) cells at time t from those at time t-Δ, in the absence of treatment. In this way it was possible to save a huge amount of computational time and to implement the model in a flexible and interactive spreadsheet program. The resulting balance equations were the following:

Np(t) = (number of proliferating cells at time t-Δ) + (newborn proliferating cells)

- (cells which had divided) + (quiescent cells entered in the proliferative status)

= Np(t-Δ) + 2·*θ*·u·Np(t-Δ)·z - u·Np(t-Δ)·z + *γ*·Nq(t-Δ)·z

Nq(t) = (number of quiescent cells at time t-Δ) + (newborn quiescent cells)

- (dead quiescent cells) - (quiescent cells entered in the proliferative status)

= Nq(t-Δ) + 2·(1 - *θ*)·u·Np(t-Δ)·z - *μ*_q_·Nq(t-Δ)·z - *γ*·Nq(t-Δ)·z

where u is given by eq. 4 and z by eq. 5.

z is close to Δ = 1 day, as *b*Δ = ln(2)·Δ/*Td *<< 1, and allows to match exactly Td of the simulation with the theoretical Td during unperturbed balanced growth. After a treatment with differential efficacy (*Sp *≠ *Sq*) the age distribution of proliferating cells will be unbalanced by quiescent cell entering the cycle (if *γ *≠ 0). In this case both u and z were approximated values, and some discrepancy of the simulation respect to a full age-dependent model is expected, for a short time after treatment. Because the interval between subsequent data points was seven days or more, this approximation can give only a small contribute to the errors of the estimate of the parameters.

The same growth equations were applied also to resistant cells (Nrp(t) and Nrq(t)).

Dying cells enter and exit three stages (d_1_, d_2_, d_3 _) of death before being lost as follows:

Nd_1_(t) = Nd_1_(t-Δ) - *k*·Nd_1_(t-Δ)

Nd_2_(t) = Nd_2_(t-Δ) + *k*·Nd_1_(t-Δ) - *k*·Nd_2_(t-Δ)

Nd_3_(t) = Nd_3_(t-Δ) + *k*·Nd_2_(t-Δ) - *k*·Nd_3_(t-Δ)

The overall number of dying-not-yet-lost cells is given by the sum of the cells in the three stages:

Nd(t) = Nd_1_(t) + Nd_2_(t) + Nd_3_(t)

The overall number of tumour cell at a time "*t*" is the sum of sensitive cycling, sensitive quiescent, resistant cycling, resistant quiescent and dying cells, namely:

N(t) = Np(t) + Nq(t) + Nrp(t) + Nrq(t) + Nd(t)

N(t) is the quantity compared with measured tumour volumes, via a proportionality constant. At the beginning of the treatment we have:

Np(0) = N(0)·*GF*·(1 - *IniR *)

Nq(0) = N(0)·(1 - *GF*)·(1 - *IniR *)

Nrp(0) = N(0)·*GF*·*IniR*

Nrq(0) = N(0)·(1 - *GF*)·*IniR*

where *GF *is the growth fraction, estimated by %Ki67+, and *IniR *represents the fraction of cells initially resistant to the drugs.

At the times of treatment, the situation immediately before (t-) is considered separately from that immediately after (t+) the treatment and the number of -surviving-cycling and quiescent cells is reduced as follows:

Np(t+) = Np(t-)·*Sp*

Nq(t+) = Nq(t-)·*Sq*

where *Sp *and *Sq *are the fraction of cells surviving the treatment, while non-surviving cells enter the first stage of dying cells:

Nd_1_(t+) = Nd(t-) + Np(t-)·(1-*Sp *) + Nq(t-)·(1-*Sq*)

When considering drug-induced resistance, the equations of surviving cells become:

Np(t+) = Np(t-)·*Sp*·(1-*Rind*)   Nrp(t+) = Nrp(t-) + Np(t-)·*Sp*·*Rind*

Nq(t+) = Nq(t+)·*Sq*·(1-*Rind*)   Nrq(t+) = Nrq(t-) + *Rs*·Nq(t+)·*Sq*·*Rind*

where *Rind *represents the fraction of – surviving – cells which become resistant as a consequence of the treatment.

The contribution of spontaneous mutations to a resistant phenotype during the 100 days of treatment was considered negligible [8].

Because the drugs were given contemporaneously, the effect of each of them cannot be evaluated separately. Thus Sp and Sq measure the effect of the combined treatment. Similarly, cells resistant to single drugs could be identified, and a single subpopulation of cells "resistant to treatment" was considered.

Because the same dosage was given each time to the patients, the same Sp and Sq were repeatedly applied on days 0, 22, 43, 64, 85, reproducing the true schedule of this study. In few instances, a more complex model was needed, shifting of the values of Sp and Sq to new Sp2 and Sq2 values in the course of the treatment.

The model is about numbers of tumour cells (N), while the data are tumour mass (V, volume), including non cancerous cells and tissues. Nevertheless, in the absence of specific information about non tumour cells (at each time and for each patient) we assumed proportionality between N and V, through the equivalence 1 cm^3 ^= 10^9 ^tumour cells. The specific value of the proportionality constant does not affect the results.

### Appendix 2. Selection of tumour growth types

In order to simplify the optimisation procedure, we fixed *Td*, *Tc*, *γ *, to representative values. This choice was justified by a preliminary study on our dataset, indicating that wide changes of these parameters only slightly modified the fits (not shown).

Representative values of the growth parameters were chosen as follows:

*Td*. Reports of doubling time of breast cancers indicate an average between 100 and 200 days, increasing with the age of the patient [34]. In the statistics of Spratt [35] only 1% have *Td *< 30 gg. Thus we considered the values *Td *= 30, 150 and 10000 days, as representative of fast, average, slow tumour, respectively.

*Tc*. The parameter represents the average length of the non-G0 part of the cycle (not to be confused with estimates of other reports [36] where quiescent cells were not considered a part). Thus Tc values usually found in cell lines *in vitro *(1–2 days) are a reasonable lower boundary. However such short Tc are not consistent in tumours with moderately high *GF*, unless accepting very high natural cell loss. We considered the values *Tc *= 2, 5 and 8 days, as representative of short, average, long cell cycle.

*γ*. The value of *γ *is in part automatically constrained by the values of the other parameters of the model of tumour growth. It is also the reciprocal of the mean residence time in the quiescent status. We consider two extreme values: 0, as representative of a tumour with negligible recycling from quiescence into the cycling stage, and 0.01, i.e. 1% quiescent cells becoming cycling per day, corresponding to an average residence time in G0 of 100 days.

Combining the values of *Td *= 30, 150, 10000 days, *Tc *= 2, 5 , 8 days and *γ *= 0, 0.01 we obtained eighteen different types (type1: *Td *= 30, *Tc *= 2, *γ *= 0; type2: *Td *= 150, *Tc *= 2, *γ *= 0; etc.) representative of tumour breast cancer growth.

For each tumour growth type, given the value of GF provided by %Ki67+, the theory [21], with cell loss only within quiescent cells, allowed to calculate additional kinetics characteristics of the tumour, namely the potential doubling time and the rate of natural cell loss, using the following formulae:

*Tpot *= (*Td*/*GF*)·(e^ln(2)*Tc*/*Td *^- 1) (derived from eq. 30 in [21])

*μ*_q _= ln(2)·(1/*Tpot *- 1/*Td *)/(1 - *GF *) (derived from eqs. 19 and 29 in [21])

*θ *= 0.5·e^ln(2)*Tc*/*Td *^- 0.5·*γ *·(2 - e^ln(2)*Tc*/*Td*^)/(*μ*_q _+ ln(2)/*Td*) (derived from eq. 15 in [21])

Some combinations of *Td*, *Tc*, *GF*, *γ *were not biologically consistent because mathematically they would require a negative cell loss. For what concerns *Tpot *we referred to the *Tpot *estimates obtained with BrdU *in vivo *in breast cancer patients by Rew and Wilson [37]. Because in that database the highest Tpot value was 50 days, we conservatively accepted a combination of parameters as biologically consistent if Tpot < 75 days.

Thus, for each patient, only a subset of the eighteen types was considered for fitting, those with Tpot > 75 days or *μ*_q _< 0 (if any) being excluded as biologically not consistent.

The data were in general poorly sensitive to the values adopted for the tumour growth parameters. In 19/35 cases, we found (Table 5) a fit statistically equivalent to the best with *Td *= 30, 150 and 10000 days. In six instances only fast growing models were compatible with the data, while in another nine fast growth was excluded.

**Table 5:** Doubling time compatible with data

**Category**	**Range**	**n**	**%**
ND	30–150–10000	19	54.3
Slow tumour	150–10000	9	25.7
Fast/intermediate	30–150	1	2.9
Fast tumour	30	6	17.1

As concerns *Tc *(Table 6), only in a minority of cases do the data indicate that two days or eight days should be preferred. In all the other cases the value remained uncertain.

**Table 6:** Cell cycle time compatible with data

**Category**	**Range**	**n**	**%**
ND	2-5-8	4	11.4
Long Tc	8	9	25.7
Intermediate/Long Tc	5–8	14	40.0
Short/Intermediate Tc	2–5	2	5.7
Short Tc	2	6	17.1

The recycling rate remained undetermined in 22/35 instances (not shown), while for the reminder the fit indicated *γ *= 0.

## Pre-publication history

The pre-publication history for this paper can be accessed here:



## Supplementary Material

Additional file 1 PAOtherapyA. Microsoft Excel program used for the simulation and fitting of tumour reduction during chemotherapy Click here for file
